# Some New Theories of Digestion

**Published:** 1895-08

**Authors:** 


### Selections and Abstracts.

---

### SOME NEW THEORIES OF DIGESTION.

*The National Medical Review.*

For a number of years, it was our privilege to be able to give almost exclusive attention to the study of physiology. Nothing can be more fascinating either to pupil or teacher. During all this time, and even up to the present day, we had to believe that "The principal object of the saliva is to moisten the food, and thus aid mastication and deglutination." And yet, we had to face the fact that an enormous quantity of saliva was secreted every twenty-four hours. It appeared almost like a waste of the forces of nature. We had to believe that the moment this saliva reached the stomach, it became inoperative. But now, all this is about to be changed. Although Freirichs came to the conclusion, a number of years ago, that salivary digestion continued in the stomach, yet his work was practically lost sight of. Now, Dr. J. H. Kellogg, of the Battle Creek Sanitarium, has just published the report of some extensive experiments in his Laboratory of Hygiene, on starch digestion. Dr. Kellogg examined the contents of the stomach, after a test meal, in 4,875 cases. In 669 of these cases, he found the starch had been completely converted into sugar. Only in 1.8 per cent. of the cases, did he find there was little or no conversion of the starch. This certainly must be accepted as conclusive, and hereafter we must teach that the digestion of starch takes place in the stomach by the aid of the saliva ferments. Clinically, this will be of great value, and must result in a number of changes in our ideas of diet.

It is only within a few weeks that a chemist of Brooklyn, New York, Prof. E. H. Bartley, published an article in the *New York Medical Journal*, setting forth the dangers of having digested starch in the stomach. Our readers may recall the fact that a number of years ago a committee of American chemists were asked to report upon the dangers of taking a pre-digested starch into the stomach. Glucose was becoming such a generally distributed article, and was so largely used in the manufacture of confectionery, that this committee was asked to report upon its effects on the system. The report was both exhaustive and conclusive, that no deleterious effects would follow its use, even in large quantities. But Prof. Bartley has recently taken exception to this report. This is a very important question, for it is a fact that to-day the best candies in the world contain a large amount of glucose; while the most popular beer on the market has recently been shown to contain a larger proportion of glucose than any other brewed in this country.

It is very interesting to analyze some of the statements of Prof. Bartley; for instance, he says that milk sugar and cane sugar are "intended" as foods in preference to grape sugar, because the former require digestion before they can be absorbed. From this, it is safe to reason that the more difficult a food is to prepare for absorption, so much the more was it "intended" as a food; therefore, boiled pork and cabbage were "intended" as foods, in preference to the more easily digested eggs and milk!

Prof. Bartley then speaks against cooked fruits, jellies, preserves, and fruit pies; because, he says, the cane sugar is changed into glucose by heating it with the acid fruits. As is well-known, "prolonged boiling" with an acid is necessary to make this change; while it is a practical fact that the housewife only brings her pears and peaches to a boil. Prof. Bartley further declares that the reason why some persons can eat raw apples "without stint, and without after-distress," and yet "cannot eat apple pie without distressing after-effects," is because the latter contains this inverted sugar! This is almost ludicrous. It occurs to us there is more difference than this between ripe raw apples and the average apple pie with its historic crust! An equally absurd illustration is where he declares, "some persons can drink lemon juice and water, but are sickened by lemonade or lemon pie." As if lemonade were cooked! For he declares that it is the heating with the acid which changes the sugar into glucose; therefore, he must always take his lemonade "after prolonged boiling!" While lemon pie, it occurs to us, has something more in it than digested starch to make it indigestible.

Prof. Bartley is evidently averse to the "sweets," for he deals the candy manufacturers a deathblow. The professor relates instances of persons who were made ill by eating candy containing this variety of sugar; and whom he restored to health by refusing them all articles containing sugar, and by giving them "pepsin and hydrochloric acid, with laxatives." This is like curing a man of some severe pain simply by combing his hair (and by the use of hypodermic injections of large doses of morphine)!

But this article is written with the view of showing how easily any number of theories may be overthrown when all the facts are made known. Prof. Bartley says that digested starch is absorbed too quickly while in the stomach, and, thereby, "may prove too great a task on the liver," and "the blood may be overcharged with dextrose." The professor reasons that when milk sugar or cane sugar is taken, it is digested below the stomach, and there more slowly absorbed. The whole drift of his article is to frighten those who take a pre-digested starch, for fear of causing diabetes!

In the light of the recent investigations of Dr. Kellogg, the absurdity of any such view is at once apparent. We now know that nature herself is digesting our starchy foods in the stomach, and that if these digested starches, or if this glucose, could in any way cause diabetes, we would, ere this, have been a race of diabetics.

For a long time, there has been a growing sentiment throughout Germany that diabetes has not been properly treated. Hirschfeld says he believes that diabetic coma is favored by the exclusion of carbonhydrates in the diet. Schmitz allows his diabetic patients a small quantity of albumen, while he orders the free use of food containing starch, and fat in large amount. Grube impregnates the system with the carbohydrates. Williamston, of Manchester, says that homemade bread is much better than especially prepared diabetic bread. A number of American physicians are following out this line of treatment with better results than they have had heretofore.

In the light of all this, we must conclude that saliva continues its action on starchy foods in the stomach until nearly, if not all, the starch is changed into glucose; that glucose is simply a normal product of digestion, and no more injurious than a digested proteid; and that the treatment of diabetes is bound to undergo a marked change in the near future.

